# Individual placement and support in Mexico: barriers and facilitators

**DOI:** 10.3389/ijph.2026.1609179

**Published:** 2026-06-10

**Authors:** J. Carlos Arámbula-Román, Elsy Cárdenas-García, Sol Durand-Arias, Ricardo Saracco-Alvarez, Jaime Carmona-Huerta, Jesús Alejandro Aldana-López, Eveling Villafuerte-Jacob, Shoshana Berenzon, Ana Carolina Florence, Bob Drake, Ezra Susser, Franco Mascayano

**Affiliations:** 1 Instituto Jalisciense de Salud Mental, Guadalajara, Mexico; 2 University Center of Health Sciences, University of Guadalajara, Guadalajara, Mexico; 3 Instituto Nacional de Psiquiatria Ramon de la Fuente Muniz, Mexico City, Mexico; 4 Columbia University, New York, NY, United States; 5 Columbia University Mailman School of Public Health, New York, NY, United States; 6 New York State Psychiatric Institute, New York, NY, United States

**Keywords:** individual placement and support, barriers, facilitators, Mexico, low- and middle-income countries

## Abstract

**Objectives:**

The Individual Placement and Support (IPS) model has proven effective in different high-income countries. However, its implementation in low- and middle-income countries presents a new challenge. This study identified potential barriers and facilitators for IPS implementation in the metropolitan area of Guadalajara, México, to prepare for a clinical trial.

**Methods:**

The study explored potential barriers and facilitators to implementing IPS in the metropolitan area of Guadalajara, Mexico, from the perspectives of 61 diverse stakeholders in mental health interest groups: policymakers, administrators, providers, service users, and potential employers. The study used semi-structured, qualitative interviews and structured questionnaires tailored to each participant group and their specific roles.

**Results:**

Major themes of barriers were stigma, limitations in treating people with serious mental illness, difficulties related to users and their support networks, and systems barriers. Potential facilitators included the effectiveness of programs and specific activities, institutional collaboration, patient-centered approach, and characteristics of IPS.

**Conclusion:**

The study provided critical information on challenges and potential strategies prior to an upcoming trial of IPS supported employment services in a large, middle-income country.

## Introduction

Serious mental illnesses (SMI) comprise a group of mental disorders that include schizophrenia, bipolar disorder, and major depression, whose nature, duration, and intensity impact life quality across social, physical, and mental health domains [1]. High unemployment consistently undermines recovery for individuals with SMI worldwide, with rates 3–6 times higher than in the general population [2].

Mexico allocates less than two percent of its health budget to mental health, with almost two-thirds of its mental health budget going to psychiatric hospitals [3]. Mexico’s investment is low, particularly for recovery-oriented services like supported employment [4]. No specific programs or laws address the employment needs of individuals with SMI. Although country-wide SMI data are not available in Mexico, a recent survey of patients with schizophrenia in Mexico City found that 71.8% were unemployed and only 5% were employed in any skilled activity [5]. Almost eighty percent of people with any disability in Mexico reported discrimination when seeking employment, and thirty percent were denied work or promotion [6, 7]. The *Instituto Jalisciense de Salud Mental* provides rehabilitation services such as psychoeducation, intermediate housing, and job workshops in Guadalajara [8], but these services are often low-intensity, do not focus on competitive employment, and lack evaluation.

In high-income countries, competitive employment provides benefits such as reduced symptoms, increased social integration, improved self-esteem, better financial management, and enhanced quality of life [9]. However, barriers like stigma, weak public policies, and limited mental health funding can restrict employment opportunities for people with SMI. These barriers may be worse in low- and middle-income countries, [10, 11].

The premier evidence-based model for SMI competitive employment, Individual Placement and Support (IPS), incorporates eight evidence-based principles: focus on competitive employment, eligibility based on client choice, attention to client preferences at every step, rapid job search, integration of rehabilitation and mental health services, personalized benefits counseling, targeted job development, and ongoing support for job maintenance [12–14]. IPS is practical, scalable, culturally sensitive, and adaptable to various settings and populations and provides individualized support for competitive employment to improve social integration [14]. A recent comprehensive review of IPS across high-income countries [13, 15] found that IPS helped two-thirds of individuals with SMI find and maintain competitive jobs, leading to its adoption and expansion in numerous high-income countries [16–21]. Nonetheless, these studies found that common barriers in high-income countries---cross-sector collaboration, lack of funding, lack of prioritization, and workforce issues---need consistent attention.

The purpose of this study was to examine current organizational, cultural, environmental, and individual barriers and potential facilitators in preparation for an IPS implementation in the metropolitan area of Guadalajara, Mexico, [22], the site of an IPS pilot trial (R34 MH131240-01).

## Methods

### Sample

This study investigated current barriers and potential facilitators of IPS implementation in Guadalajara from the perspectives of 61 interviewees in five interest groups: policymakers, clinic administrators, mental health providers, mental health service users, and potential employers. Purposive sampling identified 6 decision makers, 5 clinic administrators, 12 mental health providers, 5 adult service users at *Instituto Jalisciense de Salud Mental,* who were eligible regardless of diagnosis or employment history, consistent with the IPS principle of zero exclusion, and 33 employers in Jalisco from companies considered disability friendly. Eligibility for all groups was based on functional roles rather than demographic or clinical criteria.

### Procedures

The multidisciplinary research team, which included IPS experts, developed interview guides and questionnaires, invited eligible participants, explained the IPS model, and collected data using methods tailored to each participant group. Individual interviews were conducted with decision makers, directors, and coordinators, whose institutional roles are unique; focus groups were held with providers and users, who share similar experiences; and a structured questionnaire with closed-ended questions was administered to employers to obtain concise information on company policies and practices related to labor reintegration. Table 1 summarizes the specific details of the procedure for each group (for the complete set of interview questions, see Supplementary Material S1). All participants provided written informed consent.

**TABLE 1:** Methods used to collect information and general characteristics of the participants of the four levels of information (Metropolitan area of Guadalajara, Mexico. 2023).

Group	Method	Content of instruments	Participants
Decision makers	Semi-structured individual interviews, conducted in one session either on-site or virtually (at the convenience of the interviewee), and at times established by informants	Interviewee’s activities and roles - Knowledge about state legislation - State actions for the employment inclusion of ISMI - Views on the IPS model	Six decision-makers: Three female workers from the SNE, and three officials responsible for policies and strategies on civic participation, mental health, and inclusion (two men, one woman), between 36 and 51 years old
Clinic managers	Semi-structured individual interviews, conducted in one session either on-site or virtually (at the convenience of the interviewee), and at times established by informants	- Interviewee’s activities and roles - Patient care - Strategies and perceptions on employment inclusion - Views on the IPS model	Five key informants from *Instituto Jalisciense de Salud Mental* (four men, one woman)
Mental health providers	Semi-structured focus group interviews, conducted in one session at the workplace (or care setting) of the interviewees	- Interviewee’s activities and roles - Work environment - Strategies and experiences on employment inclusion - Views on the IPS model	Two groups of staff from *Instituto Jalisciense de Salud Mental*. The first group consisted of six women (2 psychiatrists, 2 nurses, 1 psychologist, 1 social worker). The second group included 1 male psychiatrist, 1 nurse, 2 social workers, and 2 psychologists (1 man and 1 woman)
Users	Semi-structured focus group interviews, conducted in one session at the workplace (or care setting) of the interviewees	- Opinions on received care - Personal goals and objectives - Experiences in job searching - Views on the IPS model	Five patients from *Instituto Jalisciense de Salud Mental*, diagnosed with an SMI and with more than 2 years of illness duration (four women, one man), between 34 and 49 years old
Employers	Individual structured questionnaire, conducted through the *Mentimeter* platform, consisting of 10 questions	- Company’s industry sector - Knowledge and perceptions towards SMI - Perceptions on employment inclusion of people with SMI - Views on the IPS model	33 representatives from local businesses in the metropolitan area of guadalajara, from various sectors

Interviewers described the IPS model and answered questions before all interviews. Qualitative interviews lasted approximately 40 min. For the group of potential employers, researchers presented the questionnaire in a workshop on stigma and employment challenges for patients with SMI and administered it via *Mentimeter*, an interactive software. The research team audio-recorded and transcribed Interviews and focus group discussions into *NVivo* and recorded workshop responses on *Mentimeter* and de-identified transcripts and anonymized workshop data before analysis. The *Instituto Jalisciense de Salud Mental* institutional review board approved all procedures.

### Data analysis

Using a flexible coding approach of three stages [23, 24], researchers identified *a priori* themes based on interview guides and the open-ended questions to index transcripts broadly (stage 1). Team members independently coded transcripts and then reviewed the transcripts again to identify emerging themes. During weekly videoconference meetings, they refined the codebook, incorporating both *a priori* and emerging codes (stage 2). To ensure consensus and coding reliability, they randomly selected and coded three transcripts from each interest group. Using the final codebook, researchers analyzed and organized responses into barriers and facilitators by interest group (stage 3).

## Results

Employers completed a 10-item closed-ended questionnaire assessing company characteristics, inclusion policies, prior experience hiring people with disabilities, and perceptions of SMI in the workplace. Only a minority reported current inclusion policies, and most expressed concerns about unpredictability, safety, or productivity when considering applicants with SMI. These quantitative findings are summarized in Table 2 and were integrated into the thematic analysis and are reflected within the barrier and facilitator categories presented below.

**TABLE 2:** Employer questionnaire results (Metropolitan area of Guadalajara, Mexico. 2023).

Item	Answer	N
Q1. What is your company’s line of business? And what jobs can you offer?	- Non-governmental organizations	6
	- Education and job placements	6
	- Public services	5
	- Production and manufacturing sector	5
	- Commerce and tourism	2
	- No response	10
Q2. Are you familiar with current state legislation regarding workplace inclusion?	- Yes	2
	- Partially	14
	- No	8
	- No response	9
Q3. Three words that come to mind when you think of “mental disorder”	- Negative connotations	18 12 8 9
	- Illness or health related	
	- Social or emotional connotations	
	- No response	
Q4. In your opinion, what is the employment outlook for people with severe mental illness (SMI)?	- Negative perception	19 7 2 5
	- Mixed or conditioned perception	
	- Positive perception	
	- No response	
Q5. What is the main problem you, as a company/employer, might face regarding workplace inclusion for people with SMI?	- Lack of awareness and information	16 4 4 13
	- Social stigma and prejudice	
	- Organizational challenges	
	- No response	
Q6. Does your company consider workplace inclusion for people with SMI? What actions have you taken?	- Active inclusion	7 2 1 9 2 13
	- Inclusion plans or intentions	
	- Recognition without action	
	- Non-inclusion	
	- Lack of knowledge/No definition	
	- No response	
Q7. Have you made specific agreements with people with SMI? Are there measures in place to ensure compliance with these agreements?	- Specific agreements without verification	1
	- No agreements or lack of knowledge	17
	- No response	15
Q8. How viable do you think the IPS model would be for your company? What would be the biggest challenges?	- Feasible with specific challenges	19
	- No response	14
Q9. Do you believe your company’s policies, mission, and vision are compatible with the model?	- Yes	15
	- No response or ambiguous	18
Q10. What measures do you consider relevant to facilitate/optimize the implementation of this model in your company?	- Training and awareness	11 4 3 15
	- Dissemination and communication	
	- Collaborations and organizational support	
	- No response	

Table 3 summarizes five categories of barriers: stigma, limitations in SMI care services, patient-related and support network barriers, and system barriers; and four categories of potential facilitators: actions and programs, interinstitutional collaboration, patient-centered approach, and strengths of the IPS model. Table 4 shows illustrative quotes that represent these themes.

**TABLE 3:** Analysis categories: Barriers and Facilitators. (Metropolitan area of Guadalajara, Mexico. 2023).

Barrier categories	Definition
Stigma	Experiences reported by all four levels of information when an individual with SMI seeks employment and how they perceive this process. Responses collected indicate a negative view towards people with SMI.
Limitations in SMI care services	Limitations and deficiencies identified by the first three levels of information concerning the action plan, activities, and intervention strategies for people with SMI; particularly in those actions related to labor and social integration. Within this category, three aspects are highlighted: Lack of prioritization, inadequate resources and lack of knowledge
Patient-related barriers	Responses were collected that suggest the patient also plays a responsible role in the employment inclusion process, and the problems perceived by the interviewees
Support network barriers	Communication and experiences of the first three levels of information with the support networks of the users. Responses indicated that the support network is an important part in the patient’s process
Systems barriers	Questions were raised to the initial levels of information regarding their knowledge and perceptions about laws and regulations related to mental healthcare and employment inclusion for individuals with SMI.

Facilitator categories	Definition
Actions and programs	Reach and facilitators identified by the first three levels of information about the action plan, activities, and intervention strategies intended for individuals with SMI; particularly in actions related to labor and social integration
Interinstitutional collaboration	Links and agreements (formal or informal) that the interested institutions (*Instituto Jalisciense de Salud Mental* and SNE) have with other institutions or companies for the social and/or employment integration of individuals with SMI.
Patient-centered approach	The initial levels were questioned about the attention and support they provide to meet the objectives and needs of the patients. Responses from the patients about how they perceive such attention and support were also collected
Viability of IPS	Opinions from all four levels of information about IPS, identifying its strengths and compatibilities with the current service provided

**TABLE 4:** Representative quotes of each category of analysis by stakeholders’ group (Metropolitan area of Guadalajara, Mexico. 2023).

Category/Subcategory	Group	Representative testimony
Stigma and discrimination	Decision maker/Woman from SNE, 36 years old	“The word schizophrenia or bipolarity or issues like that, people think, they see it as very intense, like someone who might have a breakdown at work, so, they shut down.”
	Clinic manager/Woman, head of dept. At SALME	“The stigma that exists, besides that, is the ignorance that exists on the part of companies to be able to hire a person who can perform their activities regardless of whether they have an SMI.”
	Provider/Male psychiatrist	“We find more intolerance and outright rejection by companies supposedly socially responsible^1^.”
	User/Woman diagnosed with SMI; 38 years old	“I didn't feel entitled sometimes to ask for leave because the schedule was very strict, or the time they deducted, the delays, and all that.”
Limitations in SMI care		
Imbalanced focus	Decision maker/Woman, director of civic participation	“There is a lack of a comprehensive health focus in the economic, housing, access to education, transportation sectors. I don’t know how this vision is with all the social determinants of health, to be able to prevent and then not require so much attention in health services.”
	Clinic manager/Man, director at SALME	“What matters to the health system is that people are not hospitalized, that they are moderately stable, and then the counterpart or what comes after that reduction in hospitalization rates, like how the person’s functionality is not within public policies, there is no link.”
	Provider/Male psychiatrist	“It’s a farm-like recovery model for mental problems and it’s a typical asylum hospital, emblematic of México’s public health policy.”
Lack of resources	Decision maker/Man, SM coordinator	“The big problem in public service is, has been, and will continue to be resources, the money.”
	Organizational/Director at SALME	“Among the main limitations are also human resources to complete the basic staff and those that cover incidents, but also to cover different shifts: Afternoon, night, weekends, with the whole staff including base psychiatrists, base general doctors, base social workers. Another limitation is the management of financial resources.”
	Provider/Female psychologist	“It also does not depend on us as workers, but on more political issues that also often create conflicts by not having all the resources.”
Lack of knowledge	Decision maker/Woman from SNE, 36 years old	“I know there are laws that prohibit discrimination, including the rights to work, to a dignified life, etc. I mean, yes, there are laws for that, but I don’t know if there are any specific norms towards mental disorders.”
​	Organizational/Director at SALME	“It happens to be difficult for a social worker, or a doctor, a psychologist, to be hugely or deeply immersed in these topics and to be able to give timely and effective follow-up.”
	Provider/Female nurse	“Most jobs, there is no recognition that the patient is feeling bad.”
Patient-related issues	Decision maker/Man, labor inclusion	“Honesty, both the family of the person and the person themselves; they do have to inform the companies what psychopharmacology, what therapy, what assistance, what specific needs they have.”
	Clinic manager/Director at SALME	“We have to work a lot with them so that they learn to be responsible, learn to tolerate frustration when something goes wrong or they are scolded, so that they don't quit at the first hurdle.”
	Provider/Female nurse	“Because of their own condition, they may not have had an average education. Many do not have secondary education, many did not finish high school, almost none have a college degree, so this poses an initial barrier to job options.”
	User/Woman diagnosed with SMI; 38 years old	“The fact of rotating shifts does not favor me much in the way that sleep is very important to me to avoid relapses. So, the time I had to rotate shifts, I couldn't handle it. I mean, I had to leave the job.”
Support network issues	Decision maker/Woman from SNE	“It’s everything: The side effects of the medications, obviously also from the diagnosis of the person, from the family, from the support they have, from the environment they are in.”
	Clinic manager/Man, sub-director at SALME	“A significant limitation I see is the lack of a support network, in fact, many times the lack of a family support network.”
	Provider/Female social worker	“We have come across relatives who are not aware that they have a disease. So, when there is no awareness of the disease, there is no support point for the patient to continue rehabilitating, basically, we have seen that the rejection is from the family itself.”
Legislative difficulties	Decision maker/Woman director of citizen participation	“The results of the initial law were not entirely good, it was not very clear how the best strategies for patient care were going to be outlined, a fully well-elaborated law was not generated, and there have been other attempts, in fact, recently there was an initiative by some political parties for the modification of the law… but we still do not have a national diagnosis, or a state diagnosis, from which the best policy strategies would have to start.”
	Clinic manager/Man, sub-director at SALME	“There are many interventions that have to be done that go beyond the health sector, the economic sector, the social sector, even the legal sector has to be involved.”
	Provider/Female psychologist	“The legal part, for example, needs to be addressed that it is not breaking a patient’s right.”
Facilitators		
Scope of strategies	Decision maker/Woman from SNE, 51 years old	“We organize all the mass events that the SNE carries out, we operate at a national level.”
	Clinic manager/Woman department head SALME	“SALME is a state reference, but it has also been a national reference in the entire care process it provides for the entire population without social security.”
	Provider/Female psychologist	“We do it multidisciplinarily, meaning I go out with the psychiatrist, the resident of the team, and social work.”
Interinstitutional collaboration and linkage	Decision maker/Man SM coordinator	“Another line I am managing from coordination is the ability to establish strategies and links with all the state universities so that students from medicine in undergraduate phases receive specific training to be able to collaborate when they enter professional activity.”
	Clinic manager/Man, director at SALME	“The reintegration program that was carried out in URI, in the comprehensive rehabilitation unit had, one, linkage with inclusive companies, two, with non-governmental organizations that allowed patients access to seek employment sources.”
	Provider/Female psychologist	“The rehabilitation unit did have this part of inclusive companies.”
Focus on the patient	System/Woman from SNE	“The job counseling is about what we are doing, about personalized attention, understanding the context in which the person is, if it’s a context of disability, understanding the limitations they present.”
	Provider/Male psychiatrist	“Provide job training to the patient linked with their family, and the family will receive a psychoeducational process from therapists that supports the environment for the patient’s rehabilitation.”
	User/Man diagnosed with SMI; 35 years old	“We are part of that, how they treat us, how they count us as equals. I guess we’re not so strange, just like normal people. I feel like being as normal as at my home, and it’s very different.”
Personal goals	Provider/Female nurse	“It’s very common for patients to express the desire to go out to work and in fact they tell you: I’m well now and I’m going to go out, I’m going to work to rent an apartment.”
	User/Woman diagnosed with SMI; 38 years old	“To be a better mother and to help me socialize more with my family, with my children and also to look for a better job.”
Viability of the IPS model	Decision maker/Woman from SNE, 38 years old	“I think it could have some applicability when the follow-up is focused and personalized.”
	Clinic manager/Man director SALME	“It’s good, because the idea is precisely that if the circumstances of each of the patients are personalized, obviously we are going to find specific support needs and even more so when a personalized follow-up is done.”
	Provider/Female social worker	“To give follow-up, especially that main thing.”
	User/Woman diagnosed with SMI; 38 years old	“It would help me to be able to work and to continue supporting and fulfilling my duties and concerns and somehow also continue organizing to have my health follow-up.”

1 In Mexico, a socially responsible company is one that, among other qualities, is supposed to maintain a commitment to recognizing people with disabilities in labor integration.

### Barrier themes

All interest groups identified stigma against serious mental illness as a pervasive barrier in Mexico; SMI care limitations, which include minimal community-based services, including supported employment: patient and family barriers, including lack of expectations for recovery; lack of legislation to promote employment of people with SMI; and lack of system and institutional coordination. The specific focus and depth of understanding differed across groups. Policy and administrative leaders emphasized that IPS reinforces their goals but also the need for public awareness and specific legislation regarding the inclusion of people with SMI. Organization directors focused on operational challenges, such as integrating health and rehabilitation services in the community. Providers described the need for increased capacity in current care settings, including the capacity to provide in-person weekly follow-ups; the need to expand community-based services that enhance functioning rather than just symptom control; and the need for family psychoeducation and inclusion. Service users emphasized stigma but also their fears regarding disclosure, concerns that available employment did not match their needs and abilities, and inadequate family support. Employers noted that only a small minority of businesses are now implementing labor inclusion measures and acknowledged their own hesitance to hire people with SMI related to widespread myths regarding the unpredictability and unemployability of people with SMI.

### Facilitator themes

All interest groups identified current efforts to promote community inclusion of people with SMI in employment as well as the need to build on current efforts to implement IPS. System leaders pointed to the need for inclusive labor legislation and the key role of the *Instituto Jalisciense de Salud Mental* as a state and national entity for providing comprehensive care to the population, emphasizing multidisciplinary actions and programs for raising awareness, sensitization, and visibility of people with disabilities that have been successfully implemented to reduce social stigma. Directors identified collaborations with multiple agencies across the country, including companies with inclusive policies, non-governmental organizations, universities, and the Human Rights Commission. Providers emphasized existing multidisciplinary teams and the importance of establishing services in the community separate from the hospital, further individualizing services, and including families. Users and families also endorsed the importance of personalized employment services that consider the person’s interests, skills, and limitations, along with dignity, empowerment, and respect. Employers noted that few businesses are implementing labor inclusion methods. They expressed mixed views on the viability of the IPS model but emphasized their interest in individualized follow-up and personalized support as positive aspects of IPS.

## Discussion

### Summary

This study identified five themes regarding barriers to IPS implementation and four regarding potential facilitators. Major barrier themes were cultural stigma, limitations in community-based services for people with serious mental illness, minimal understanding of community-based services and satisfactory job matches among users and their support networks, and lack of coordination among systems of healthcare and employment. Potential facilitators involved enhancing the effectiveness of programs, improving legislation to promote disability rights, increasing specific activities such as including families, improving institutional collaboration, emphasizing a patient-centered approach, and incorporating the basic principles of IPS into services.

### Interpretation

Jalisco is relatively early in the process of deinstitutionalization and, despite progress, is still shifting resources to community-based services that promote opportunities for community integration of people with SMI. Stigma and myths regarding unemployability remain strong, although community leadership, adequate funding of services, and collaboration among agencies are in development. Basic principles of IPS, such as a belief that everyone can work, a focus on individualized job matches and supports, cross-sector collaborations, and education for families and employers, could enhance positive change.

### Comparisons with previous research

Interviewees in Jalisco identified many barriers that resemble those reported in high-income countries, such as financing limitations, systems barriers (e.g., public policies or employment issues), and workforce issues (e.g., better pay for IPS specialists) [17–21]. Nevertheless, Mexico is a middle-income country rather than a high-income country with a largely hospital-based mental health system. Greater poverty and the lack of community-based services make a difference. Due to resource limitations and the stage of mental health reform, identified barriers in Mexico were more extreme and challenging than those in high-income countries.

Identified facilitators were also similar to changes that other countries have faced: e.g., continuing to develop leadership, funding, quality improvement, government actions and programs, and close collaboration between mental health and IPS agencies, regular fidelity reviews, technical assistance, person-centered services, collaboration with families, training of mental health staff, and educating employers regarding the advantages of hiring people with disabilities. At the time of this study, most of these changes were underway in Jalisco. Instantiating the principles of IPS may reduce stigma and enhance several positive trends.

IPS has been adapted for many new populations and new countries [13, 15], but the current project in Jalisco is the first to consider adaptations in Mexico. Success may require strengthening anti-stigma efforts and changes in local laws, customs, and approaches to health and employment services as well as modifications to the IPS model. Shifting resources to community-based services and further collaboration with employers may be critical. Extensive evidence on interventions against stigma exists [25], including interventions targeting social participation [26]. The IPS implementation could potentially address close contact, raising awareness among employers, mitigating self-stigma through the participation of support networks, and creating greater visibility towards legislative actors. Currently, *Instituto Jalisciense de Salud Mental* is trying to implement strategies in this regard, but the lack of resources limits---one of the main challenges in developing Mexico’s mental health system---limits reach [27].

These findings provide practical guidance for the forthcoming IPS pilot in Guadalajara. The qualitative themes highlight specific system-level obstacles—such as fragmentation across agencies and limited community-based services—as well as employer concerns related to safety, predictability, and readiness. Identifying these issues in advance allows the implementation team to anticipate challenges, target employer education, strengthen inter-agency collaboration, and plan supports for individualized job matching. In this way, the qualitative approach used in this study directly informs pre-implementation planning and establishes priorities for adapting organizational processes during the initial rollout of IPS.

### Limitations

Respondents lacked actual experience with IPS and were therefore expressing impressions and attitudes toward an unfamiliar intervention that has worked well in other countries, cultural contexts, and social, health, and economic systems but has not been tried in Mexico. This sampling strategy was intentionally designed for an exploratory pre-implementation phase, in which the goal is to obtain perspectives from key institutional stakeholders directly involved in the mental-health and employment systems in Guadalajara rather than to achieve statistical representativeness. For this reason, eligibility was based on functional roles and institutional relevance rather than demographic or clinical criteria. Broader sampling may be appropriate for future studies focused on statewide or national scaling. In addition, convenience sampling may have introduced unknown biases that misrepresent the actual views of introducing IPS in Jalisco.

### Conclusions

This analysis prior to IPS implementation provided a preliminary understanding of the barriers and facilitators within the local context, according to different interest groups. The study may enable more effective planning for the initial IPS trial in México. By implementing IPS at this stage of developing community-based services, Mexico could bypass many of the ineffective interventions, such as day treatment and sheltered workshops, that have delayed IPS progress in other countries.
