# Supplementary material for: Individual placement and support in Mexico: barriers and facilitators
Source: Int J Public Health. 2026 Jun 10;71:1609179. doi: 10.3389/ijph.2026.1609179 (PMC13290708; doi:10.3389/ijph.2026.1609179)
Supplement: Supplementary file 1 [file DataSheet1.docx]

**Supplemental File 1. Interview guides (Metropolitan area of Guadalajara, Mexico. 2023).**

| **Group** | **Interview questions** |
| --- | --- |
| Decision Makers | 1. Could you tell us about the current state legislation on the subject of mental health? What is being done for people with these conditions? 2. Do you think this is enough to cover the needs of people with SMI? 3. What is the main problem facing the State regarding mental health issues? 4. Regarding discrimination in mental health, what has been done about it? Is there an action plan to promote inclusion? 5. Is labor inclusion taken into consideration for people with SMI? What does the law say about it? 6. What is the panorama on the employability of those people who suffer from mental disorders, particularly with people whose social functionality is affected? 7. Are there specific policies that have been agreed between employers and people with SMI? Are steps taken to verify compliance with the agreement? 8. In your opinion, how do you see the relationship between the labor market in Jalisco and people with SMI? Do you think there are good opportunities? 9. What are the challenges that the State currently faces regarding the formal employability of people with SMI? 10. How viable do you think it would be applicable in Jalisco? 11. Do you think that the current mental health system and the model are compatible? what would be the biggest challenges? 12. What measures do you consider pertinent to facilitate/optimize the implementation of this model in the state/Guadalajara Metropolitan Area (GMA)? |
| Clinic Managers | 1. In general, what does the SALME action plan consist of? 2. What is the situation regarding patients with SMI? (e.g., scope and limitations that have been or have). 3. What does SALME consider to be the ultimate goal in the treatment of these patients? Do you think it is being achieved? 4. Do you think this meets the needs of people with SMI? 5. There are proposals that mention that social reintegration can be achieved through labor insertion or reinsertion. What is SALME's position on the matter? 6. Does SALME implement strategies that support or guide users to obtain or maintain a job? 7. What do you think of the model/project? 8. Do you think that the current mental health system and the IPS model are compatible? 9. Do you think it is possible to implement the IPS model in SALME? Why? 10. How can SALME facilitate/optimize the implementation of IPS? |
| Mental Health providers | 1. Please tell us about your experience with patients within the organization. 2. What are the activities that you carry out as mental health providers? 3. If it were up to you, would you modify the intervention program? If so, how? 4. Do you have experience regarding the labor reintegration of patients who have suffered a SMI? Do you use any model or intervention to support them in obtaining employment? 5. Regarding the previous question, what have been the challenges you have overcome? What is still a limitation to overcome? 6. How do you consider the organizational climate in which you work? 7. What do you think of the model/project? 8. As mental health professionals and providers, do you think you have the necessary skills to implement the model? 9. Do you think that the state of the current organizational climate is sufficient to be able to implement the IPS model in this institution? 10. The IPS implies strong communication between teams of psychiatry, social work and psychology. How do you consider the current level of communication of the institution between these teams? 11. Personally, do you consider the application of the IPS viable? 12. What strategies do you consider pertinent to optimize or facilitate the implementation of IPS? |
| Users | 1. What is your opinion of the general care provided by mental health services? 2. Do you have any particular goals that you would like to achieve? 3. What do you think about going back to work? (or start working, in any particular case). 4. Do you think that being here helps you meet your goals? 5. What do you think about employment opportunities for people living with SMI? What has been your experience? 6. Have you been offered any kind of government assistance to help you find a job? 7. What do you think of the model/project? 8. Do you think that the model is useful for you to get a job? why? 9. In what way do you think you could be helped to find a job more easily? |
| Employers | 1. What is your company's line of business? And what jobs can you offer? 2. Are you familiar with current state legislation regarding workplace inclusion? 3. Three words that come to mind when you think of "Mental Disorder" 4. In your opinion, what is the employment outlook for people with severe mental illness (SMI)? 5. What is the main problem you, as a company/employer, might face regarding workplace inclusion for people with SMI? 6. Does your company consider workplace inclusion for people with SMI? What actions have you taken? 7. Have you made specific agreements with people with SMI? Are there measures in place to ensure compliance with these agreements? 8. How viable do you think the IPS model would be for your company? What would be the biggest challenges? 9. Do you believe your company's policies, mission, and vision are compatible with the model? 10. What measures do you consider relevant to facilitate/optimize the implementation of this model in your company? |
